# Grass carp SERPINA1 inhibits GCRV infection through degrading CF2

**DOI:** 10.3389/fimmu.2022.969517

**Published:** 2022-09-08

**Authors:** Yangyang Li, Liangming Chen, Rong Huang, Yangyu Li, Cheng Yang, Bin Gui, Yongming Li, Lanjie Liao, Zuoyan Zhu, Yaping Wang

**Affiliations:** ^1^State Key Laboratory of Freshwater Ecology and Biotechnology, Institute of Hydrobiology, Chinese Academy of Sciences, Wuhan, China; ^2^University of Chinese Academy of Sciences, Beijing, China; ^3^Innovative Academy of Seed Design, Chinese Academy of Sciences, Beijing, China

**Keywords:** SERPINA1, CF2, GCRV, overexpression, virus suppression

## Abstract

SERPINA1, a member of the serine protease inhibitor family, plays a role in viral infection and inflammation by regulating the activities of serine and cysteine proteases. To date, there have been no reports on the immune function of SERPINA1 in fishes. In this study, we first cloned the *serpina1* gene of grass carp (*Ctenopharyngodon idellus*) and found that it could respond rapidly to the infection of Grass carp reovirus (GCRV), and overexpression of *serpina1* could enhance the antiviral response of CIK cells. A polyclonal antibody of SERPINA1 was prepared, and the protein interacting with SERPINA1 was screened by CoIP/MS in grass carp hepatopancreas tissue. It was found that SERPINA1 interacted with coagulation factor 2 (CF2) and could degrade it in a dose-dependent manner. In addition, overexpression of *cf2* contributed to the infection of GCRV in CIK cells, whereas co-expression of *serpina1* and *cf2* in grass carp reduced the copy number of GCRV in cells. The results showed that grass carp SERPINA1 could inhibit GCRV infection by degrading CF2. This study proposes that SERPINA1 can inhibit viral infection through interaction with the coagulation factor, providing new insights into the molecular mechanism of SERPINA1’s antiviral function.

## Introduction

Grass carp (*Ctenopharyngodon idellus*) is the most abundant fish species in freshwater aquaculture worldwide, with an annual output of 5.704 million tons in 2018, accounting for 10.5% of the world’s major aquaculture species (1). However, grass carp are extremely vulnerable to grass carp reovirus (GCRV), which causes hemorrhagic disease, leading to a mortality rate as high as 90% (2, 3). This has caused enormous economic losses to the grass carp farming industry (4).

The serine proteinase inhibitor (SERPIN) gene family is the largest protease inhibitor gene family in organisms, with many members known for their extensive inhibition of serine proteases (5, 6). Members of the SERPIN family exhibit high sequence similarity and functional diversity (7–10). There are 16 evolutionary branches of mammalian SERPINs and one of the largest subfamilies is SERPINA. Previous studies have demonstrated that SERPIN genes are involved in the antiviral immune process. The degree of inflammation increases in mice with *serpinb1* or *serpinb6* deficiency (11, 12). In addition, *serpinc1* has antiviral activity against HIV, HCV, and HSV, inducing PTGS2 to inhibit HIV-1 virus copies in PBMC (13). The relationship between SERPIN and viral infection is one of the hottest research directions for antiviral therapy (14).

SERPINA1 of the SERPIN family is commonly known as alpha-1 antitrypsin (AAT) (15). Previous studies have shown that SERPINA1 has anti-inflammatory activity and can be quickly triggered in response to inflammation in the body (16–19). It can also reduce the production of pro-inflammatory factors such as TNF-α and IL-8 (20, 21). During the process of viral infection, SERPINA1 inhibits HIV-1 replication in U1 monocytes (22). SERPINA1 can suppress the replication of H1N1 virus in primary rhesus monkey kidney cells and significantly reduce the baseline of inflammatory cytokines in mice (23). To date, immune function of the fish *serpina1* gene has not been reported.

Our research team is committed to study the disease resistance-related immune mechanisms in grass carp, focusing on the identification of immune response-related genes involved in the process of GCRV infection. In a previous study, several immune response genes involved in GCRV infection were identified in grass carp using transcriptome sequencing, including the *serpin* genes (24). This study is focused on SERPINA1, which belongs to the SERPINA subfamily. By screening the proteins interacting with SERPINA1 during GCRV infection, we determined whether SERPINA1, along with related proteins, can affect GCRV replication. This study broadens the relationship between the serine protease inhibitor family and GCRV infection in grass carp and analyzes the possible mechanisms through which SERPINA1 suppresses viral infection *in vivo*.

## Materials and methods

### Grass carp, cells, virus, and ethics

This study used 6-month-old grass carp (15 ± 3 cm, 40 ± 10 g) provided by the Guanqiao Experimental Base of the Institute of Hydrobiology, Chinese Academy of Sciences. Human embryonic kidney (293T) cells (China Center for Type Culture Collection, CCTCC) were cultured at 37 °C in 5% CO_2_ in Dulbecco’s modified Eagle’s medium (DMEM; Invitrogen), supplemented with 10% fetal bovine serum (FBS; Invitrogen). CIK cells (CCTCC) were maintained at 28 °C in 5% CO_2_ in Medium 199 (M199; Invitrogen). The GCRV was prepared and preserved in our laboratory (25). The feeding, sampling, and virus infection experiments for all experimental fish were approved by the Academic Committee of the Institute of Hydrobiology, Chinese Academy of Sciences (CAS) (Y32A011).

### Cloning, sampling, and qPCR detection of *serpina1*

The zebrafish *serpina1* sequence (NM_001077758.1) was obtained from the NCBI website, aligning the sequence with the grass carp genome (26) to obtain the coding sequence (CDS) of grass carp *serpina1* (ON868913). Specific primers were designed for 5’ RACE and 3’ RACE following the SMARTer RACE 5’/3’ Kit instructions to amplify fragments. The amplified fragments were sequenced and spliced to obtain the full-length cDNA sequence of the grass carp *serpina1*. All primers designed for gene cloning and sequence verification were listed in **Supplementary Table 1**.

The brain, kidney, head kidney, spleen, intestine, heart, gill, and hepatopancreas tissues were taken from healthy 6-month-old grass carp (n=6). The remaining fish were infected intraperitoneally (n=100) with GCRV virus solution (3.57×10^7^ TCID_50_/ml) at a dose of 2% (vol/g) of their body weight. The head kidney, spleen, gills, intestine, and hepatopancreas were sampled after the attack (n=6). The sampling time included six consecutive days after the attack and days 8, 11, and 14. Samples were collected randomly during the incubation period (before day 8) and the onset period (day 8 and onwards) for grass carp with significant symptoms. The samples were ground in TRIzol (Invitrogen, Carlsbad, CA, USA) and stored at -80°C until use.

The tissue samples were extracted using TRIzol reagent (Invitrogen, Carlsbad, CA, USA), and cDNA was synthesized using the EasyScript^®^ All-in-One First-Strand cDNA Synthesis SuperMix for qPCR (One-Step gDNA Removal) (TransGen Biotech. Beijing, China). The primers used for qPCR are listed in **Supplementary Table 1**. The *β-actin* was used as an internal control. Relative expression level was calculated using the 2^-ΔΔCt^ method (27) and expressed as mean ± standard deviation. Three independent experiments were conducted for statistical analyses. Data were processed using SPSS25 statistical software and assessed for significant differences using the Student’s *t*-test.

### Antibody preparation of SERPINA1

The CDS region of grass carp *serpina1* was subcloned into the pEASY-Blunt vector (TransGen Biotech, Beijing, China) and then transformed into *E. coli* BL21 (DE3) to generate recombinant SERPINA1. The expression was induced at 37 °C and 1 mM IPTG, purified with Ni-NTA affinity medium, and the gradient concentration of imidazole (40, 80, 120, 160, and 200 mM) was used for elution. Japanese white rabbits were immunized according to the routine method of the Animal Experimental Center of the Wuhan Institute of Virology, Chinese Academy of Sciences. Before immunization, 2 mL of blood was collected from the ear vein to prepare negative control serum, then the hosts were immunized with 400 μg SERPINA1 protein for the first time, thereafter with 450 μg SERPINA1 protein on day 14 and day 28 after initial immunization. The supernatant of rabbit whole blood was isolated as SERPINA1 polyclonal antibody 14 days after the second booster immunization.

### Sample preparation for screening SERPINA1 interacting proteins

SERPINA1 was detected to have a high expression level in grass carp hepatopancreas tissue. As a result, samples of hepatopancreas tissues from grass carp (n=3) before (healthy) and 8 days after the infection (post-infection) were selected. Grass carp were infected by intraperitoneal injection. The total tissue proteins were extracted according to the manufacturer’s instructions for cell lysate (Beyotime, Shanghai, China). The types and abundance of proteins bound to SERPINA1 before and after the infection were detected and analyzed using CoIP-MS.

The experimental procedure was as follows: 250 μL SERPINA1 antibody (1:1,000 dilution) was added to the total protein (500 μL) of grass carp hepatopancreas tissue (healthy and post-infection tissue). In the control groups, SERPINA1 antibody was replaced by 250 μL negative control serum (1:10,000 dilution), and both the groups were incubated at 4°C overnight. Thereafter, 50 μL protein A+G agarose (Beyotime, Shanghai, China) was added to the experimental and control groups and were kept for incubation at 4°C for 4 h. The supernatant was removed by centrifugation and the precipitate was washed with 1 mL cell lysate. The supernatant was used as the eluted protein sample after instantaneous high-speed centrifugation for mass spectrometry analysis.

### Proteins mass spectrometry analysis

The eluted protein samples were precipitated and washed with TCA. 8 M Urea (dissolved in 100 mM Tris-HCl solution, pH 8.0) was added to the protein precipitate, and the supernatant was obtained by centrifugation. DTT was added to a final concentration of 10 mM and incubated at 37 °C for 1 h. Thereafter, IAA was added to 40 mM for alkylation. Finally, 100 mM Tris-HCl solution (pH 8.0) was added before using Bradford’s method to quantify the protein concentration, which was processed for sequencing according to the manufacturer’s instructions (28).

Protein peptide sequencing was performed using a TripleTOF 5600+ liquid mass spectrometry system (Applied Biosystems/MDS SCIEX, Foster City, CA, USA) following the manufacturer’s instructions (28). For mass spectrometry DDA mode analysis, each scan cycle contained one full MS scan (scan range: 350–1500 m/z, ion accumulation time: 250 ms) and 40 subsequent MS/MS scans (scan range: 100–1500 m/z, ion accumulation time: 50 ms). MS/MS scans were triggered by peptide ions with signals above 120 cps (+2 to +5). The exclusion time for MS/MS repeat acquisition was set at 18 s. The generated mass spectrometry data were retrieved by ProteinPilot (V 4.5) using the Paragon retrieval algorithm (29) and our laboratory’s grass carp proteome reference database. Spectra files were searched against the target database using the following parameters: Sample Type: Identification, Cys Alkylation: Iodoacetamide, Digestion: Trypsin/P, and Search Effort: Rapid ID. Search results were filtered with Unused ≥1.3. Proteins denoted as decoy hits or contaminants were removed and the remaining proteins were used for further analysis. Based on the MS/MS counts among different sample groups, significant proteins were obtained.

After removing the proteins from the control samples for each of the healthy and post-infection immunoprecipitation samples, data with a spectral count of 0 were filled with 1 for statistical analysis. For the identification of expression differences, each experimental run was initially considered separately. The ratio of the spectral counts (ratio) and their mean (MeanSP) is the vertical coordinate scatter plot. The t-test was performed on the spectrogram number of each sample protein before and after the infection, and when the protein spectrogram number ratio difference exceeded 1.5-fold (spectrogram number tends to +∞) to 3-fold (spectrogram number tends to 1) with *p* ≤ 0.05, the protein was labeled as “++/–”; otherwise, it was labeled as differential protein (“+/-”).

### Coagulation factor 2 (CF2) cloning and validation of SERPINA1 and CF2 interaction

Mass spectrometry-based sequencing analysis identified CF2 as a candidate protein for SERPINA1 binding. The full-length of the grass carp *cf2* gene was obtained from the grass carp genome (26) based on the ID number of the gene (CI01000026_10831971_10837028). The CDS region of the grass carp *cf2* gene (ON868914) was subcloned into pCMV-Myc to construct the eukaryotic expression plasmid, pCMV-Myc-*cf2*. The CDS region of the grass carp *serpina1* gene was subcloned into pCMV-HA to construct the eukaryotic expression plasmid pCMV-HA-*serpina1*. The cloning primers used are listed in **Supplementary Table 1**.

Furthermore, 293T cells seeded into 10 cm^2^ dishes overnight were co-transfected with 4 μg of pCMV-HA-*serpina1* and pCMV-Myc-*cf2* each using Lipo6000 (Beyotime, Shanghai, China), and the cells were collected after 24 h. Total protein was extracted from the cells using a cell lysate. Thereafter, 500 μL of total protein was added to 250 μL of SERPINA1 antibody and incubated overnight at 4 °C. The supernatant was removed by centrifugation, and 1 mL of the cell lysate was washed and subjected to SDS-PAGE. Immunoprecipitants or whole-cell extracts were transferred onto polyvinylidene difluoride (PVDF) membranes (Bio-Rad, Berkeley, CA). The membranes were blocked for 1 h at 25 °C in TBST buffer (25 mM Tris-HCl, 150 mM NaCl, 0.1% Tween 20 [pH 7.5]) containing 5% nonfat dry milk, probed with the anti-Myc Ab (Proteintec, Rosemont, USA) or the anti-HA Ab (Proteintec, Rosemont, USA) at an appropriate dilution overnight at 4°C, washed three times with TBST, and then incubated with HRP-conjugated anti-rabbit IgG for 1 h at room temperature. After three additional washes with TBST, the membranes were stained with the Immobilon ImageQuant LAS 4000 system (GE Healthcare). The antibodies were diluted as follows: anti-*β*-actin (ABclonal Technology, Woburn, MA) at 1:1000, anti-Myc/HA (Proteintec, Rosemont, USA) at 1:3000, and HRP-conjugated anti-rabbit IgG (Sigma Aldrich, St. Louis, USA) at 1:5000.

In addition, co-transfection experiments with different concentrations of the gradient plasmids were performed. Thereafter, 4 μg pCMV-Myc-*cf2* was transfected with 0, 2, 4, and 8 μg of pCMV-HA-*serpina1* plasmids, and the total amount of transfected plasmids was increased to 12 μg using empty pCMV-Myc plasmids. After 24 h, total cell protein was extracted, protein A+G agarose was added, CoIP experiments were performed as described above, and protein immunoblotting was performed.

### SERPINA1 and CF2 fluorescence confocal analysis

The grass carp *serpina1* CDS region was subcloned into pEGFP-N3 to construct the green fluorescent fusion expression plasmid, pEGFP-N3-*serpina1*. The grass carp *cf2* CDS region was cloned into pCS2-mCherry to construct the red fluorescent fusion expression plasmid, pCS2-mCherry-*cf2*. The cloning primers used are listed in **Supplementary Table 1**. CIK cells were plated onto coverslips in six-well plates, transfected with 2 μg of pEGFP-N3-*serpina1* or pCS2-mCherry-*cf2* plasmids, and co-transfected with pEGFP-N3-*serpina1* and pCS2-mCherry-*cf2* (2 μg each) using Lipo6000; control groups were co-transfected with pEGFP-N3-*peli2* (GFP-tagged irrelevant protein) and pCS2-mCherry-*cf2* (2 μg each). After 24 h of transfection alone, and 24 and 48 h of co-transfection, the cells were washed with PBS, fixed by adding 1 mL of 4% paraformaldehyde for 15 min, and stained with 1 mL Hoechst 33242 (Beyotime, Shanghai, China) for 10 min in the dark at room temperature. Finally, the coverslips were washed and observed using a laser confocal microscope (PerkinElmer, Fremont, CA, USA) under a 63× oil-immersion objective.

### Effects of *serpina1* and *cf2* individual overexpression and co-expression on GCRV infection

The CIK cells were seeded in six-well plates, transfected with 2 μg of pCMV-HA-*serpina1* or pCMV-Myc-*cf2* plasmids, and co-transfected with pCMV-HA-*serpina1* and pCMV-Myc-*cf2* (2 μg each) using Lipo6000. The control groups were transfected with equal amounts of empty plasmids pCMV-HA or pCMV-Myc. The GCRV infection assay was performed 24 h later. The infection assay procedure involved the addition of 1 mL of laboratory-prepared GCRV virus solution (3.57×10^7^ TCID_50_/ml) to the cells, and 1 mL of serum-free M199 was added to the control group. All the culture media were replaced with serum-free M199 medium after 2 h of viral incubation. The cells were collected after 24 h, and 1 mL of TRIzol was added to lyse the cells. The cDNA template was prepared as described in Section 2.2, and the mRNA expression levels of inflammatory factors (*tnf-α*, *il-6*, *il-8*, and *il-10*), antiviral genes (*mx2*, *pkr*, *viperin*, and *ifn1*), and GCRV *s6* fragments were detected through qPCR.

For co-transfection experiments, CIK cells were seeded in six-well plates and then transfected with 2 μg of pCMV-Myc-*cf2* along with 0, 0.5, 1, and 2 μg of pCMV-HA-*serpina1*, respectively. They were all supplemented to 4 μg using the empty pCMV-Myc plasmid to ensure that the total amount of DNA transfected was uniform in each well. The cell samples were collected 12 and 24 h after GCRV infection. The cDNA templates were prepared as described in Section 2.2, and qPCR was used to detect changes in GCRV *s6* expression in the cell samples from different experimental groups.

## Results

### Characteristics of grass carp *serpina1* and changes in response to GCRV infection

**Figure 1:**
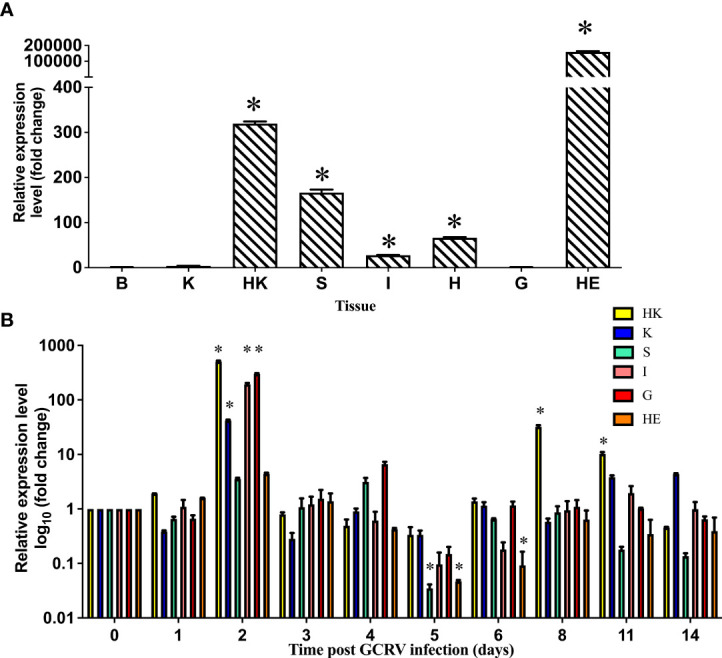
Expression distribution of *serpina1* mRNA in various tissues of grass carp before and after infection. **(A)** Expression distribution of *serpina1* mRNA in the brain (B), kidney (K), head kidney (HK), spleen (S), intestine (I), heart (H), gill (G), and hepatopancreas (HE) of healthy grass carp. The expression level of the *serpina1* gene in the brain was taken as “1”, and the relative expression levels of other tissues were calculated. The horizontal coordinates indicate the different tissues, and the vertical coordinates indicate the relative expression. **(B)** Changes in the expression of *serpina1* mRNA in the HK, K, S, G, I, and HE of grass carp after GCRV infection. The expression before infection (day 0) was taken as “1”, and the relative expression levels of the *serpina1* gene after infection were calculated. The horizon coordinates indicate different infection times, and the vertical coordinates indicate the relative expression level. Different colors indicate different tissues. “*” indicates p<0.05.

The cDNA sequence of the grass carp *serpina1* gene consisted of 1,329 bp CDS sequence and 132 bp 5′-end non-coding sequence, and 246 bp 3′-end non-coding sequences. The expression characteristics of the *serpina1* gene were analyzed in eight healthy grass carp tissues using qPCR (brain, kidney, head kidney, spleen, intestine, heart, gill, and hepatopancreas) (**Figure 1A**). As shown in the figure, the gene had the highest expression level in hepatopancreas tissues; lower expression levels in the brain, kidney, and gill; and moderate expression levels in the head kidney, spleen, intestine, and heart. The highest expression level (hepatopancreas) differs 200,000 times from the lowest expression level (brain).

Furthermore, qPCR analysis of the *serpina1* response in the head kidney, kidney, spleen, gill, intestine, and hepatopancreas before and after GCRV infection was performed (**Figure 1B**). As shown in the figure, the response of *serpina1* was obvious in the head kidney, kidney, intestine, and gill, with peak expression on day 2 after infection (506.5-fold, 41.8-fold, 195.2-fold, and 296.2-fold, respectively; *p* < 0.05), and then gradually decreased and returned to normal levels by day 14. In contrast, the expression of *serpina1* in the spleen and hepatopancreas was relatively weak, first slowly increasing and then slowly decreasing, reaching its lowest expression (0.04-fold and 0.05-fold, respectively; *p* < 0.05) on day 5 after infection and then gradually increasing to normal levels by day 14.

### Mass spectrometry analysis identified CF2 as a possible protein interacting with SERPINA1

**Figure 2:**
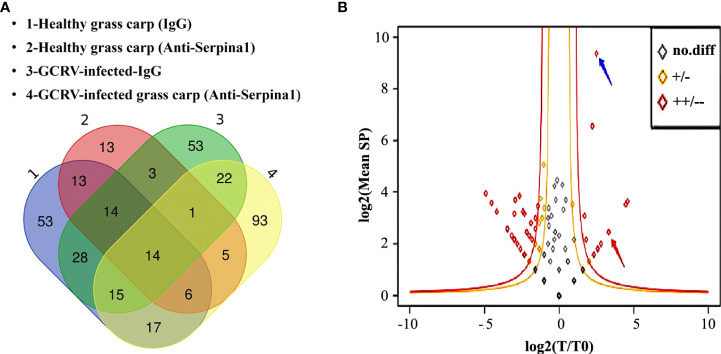
Screening of differentially bound proteins in mass spectrometry analysis. **(A)** Venn diagram of immunoprecipitated samples before and after the GCRV infection containing total protein classes. “1” represents negative control serum co-precipitated with healthy grass carp hepatopancreas tissue samples; “2” represents SERPINA1 antibody co-precipitated with healthy grass carp hepatopancreas tissue samples; “3” represents negative control serum co-precipitated samples with post-infected grass carp hepatopancreas tissue, and “4” represents SERPINA1 antibody co-precipitated samples with post-infected grass carp hepatopancreas tissue. **(B)** Scatter plots of differentially bound proteins. The outer red line is the highly significant difference interval. The blue arrows point to SERPINA1, and the red arrows point to CF2. The horizontal coordinates indicate the logarithm of the ratio of SERPINA1 antibody to the number of protein profiles in the hepatopancreas tissues of grass carp before and after GCRV infection. The vertical coordinates indicate the logarithm of the mean number of protein spectral counts in each sample.

SERPINA1 antibody was tested by western blot assay with a titer of 1:1,000 dilution (**Supplementary Figure 1**). After preparation of polyclonal antibodies against grass carp SERPINA1, immunoprecipitation experiments were performed with healthy and GCRV-infected hepatopancreas tissues from grass carp, and the immunoprecipitated proteins were subjected to MS full scan to collect peptide data and search the grass carp proteome database. A total of 614 peptides corresponding to 160 proteins were detected by SERPINA1 antibody and healthy hepatopancreas tissue co-precipitation samples; 226 peptides corresponding to 70 proteins were detected in negative control serum and healthy hepatopancreas tissue co-precipitation samples; 748 peptides corresponding to 150 proteins were detected by SERPINA1 antibody and GCRV-infected hepatopancreas tissue co-precipitation samples; and 863 peptides corresponding to 224 proteins were detected in negative control serum and GCRV-infected hepatopancreas tissue co-precipitation samples. (**Figure 2A**)

Scatter plots of the differential proteins before and after GCRV infection were plotted after removing the proteins contained in the control samples from each of the healthy and GCRV infection immunoprecipitation samples (**Figure 2B**). A total of 56 differentially expressed proteins (red and orange dots) were found, among which three proteins were increased (*p* > 0.05), 13 proteins were significantly increased (including SERPINA1 itself) (*p* ≤ 0.05), 13 proteins were decreased (*p* > 0.05), and 27 proteins were significantly decreased (*p* ≤ 0.05). Twelve proteins were significantly increased in the binding of SERPINA1 antibody to grass carp hepatopancreas tissue before and after the infection (**Supplementary Table 2**). CF2 is a precursor of thrombin (30). Since GCRV can cause grass carp to present hemorrhagic symptoms, among these highly significant differential proteins, the associated coagulation and anticoagulation factor proteins are of great interest; therefore, a follow-up analysis for CF2 was conducted.

### CoIP demonstrates dose-dependent interaction of SERPINA1 and CF2

**Figure 3:**
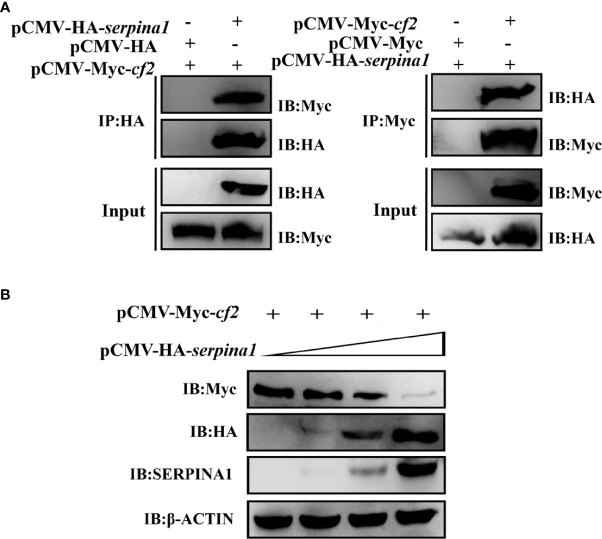
*In vitro* cellular assay demonstrating the interaction between SERPINA1 and CF2 in a dose-dependent manner. **(A)** CoIP verifies that SERPINA1 and CF2 interact with each other in 293T cells. “IP” indicates immunoprecipitation experiments with affinity samples incubated with HA or Myc antibodies, and “IB” indicates immunoblotting experiments with HA or Myc antibodies. **(B)** Western blot to detect the degradation of CF2 by SERPINA1. “IB” indicates immunoblotting experiments on protein samples with HA, Myc, SERPINA1, or β-ACTIN antibodies.

Plasmids expressing SERPINA1 and CF2 were co-transfected into 293T cells, and CoIP was used to verify the interaction between SERPINA1 and CF2. The results showed that protein bands of both proteins were detected in the co-precipitated samples (**Figure 3A**), regardless of whether the CoIP experiments were performed with the tag antibody of SERPINA1 (HA Abs) or with the tag antibody of CF2 (Myc Abs), indicating that there is indeed an interaction between grass carp SERPINA1 and CF2. In addition, while expressing a fixed amount of CF2 protein in 293T cells (plasmid transfection amount fixed at 4 μg), the expression of SERPINA1 protein gradually increased (plasmid transfection amount 0, 2, 4, and 8 μg). The results showed that the protein expression of SERPINA1 gradually increased, whereas the protein amount of CF2 gradually decreased (**Figure 3B**), indicating that SERPINA1 can degrade CF2, and that the degree of degradation was dose-dependent.

### Fluorescence confocal confirms degradation of CF2 by grass carp SERPINA1

**Figure 4:**
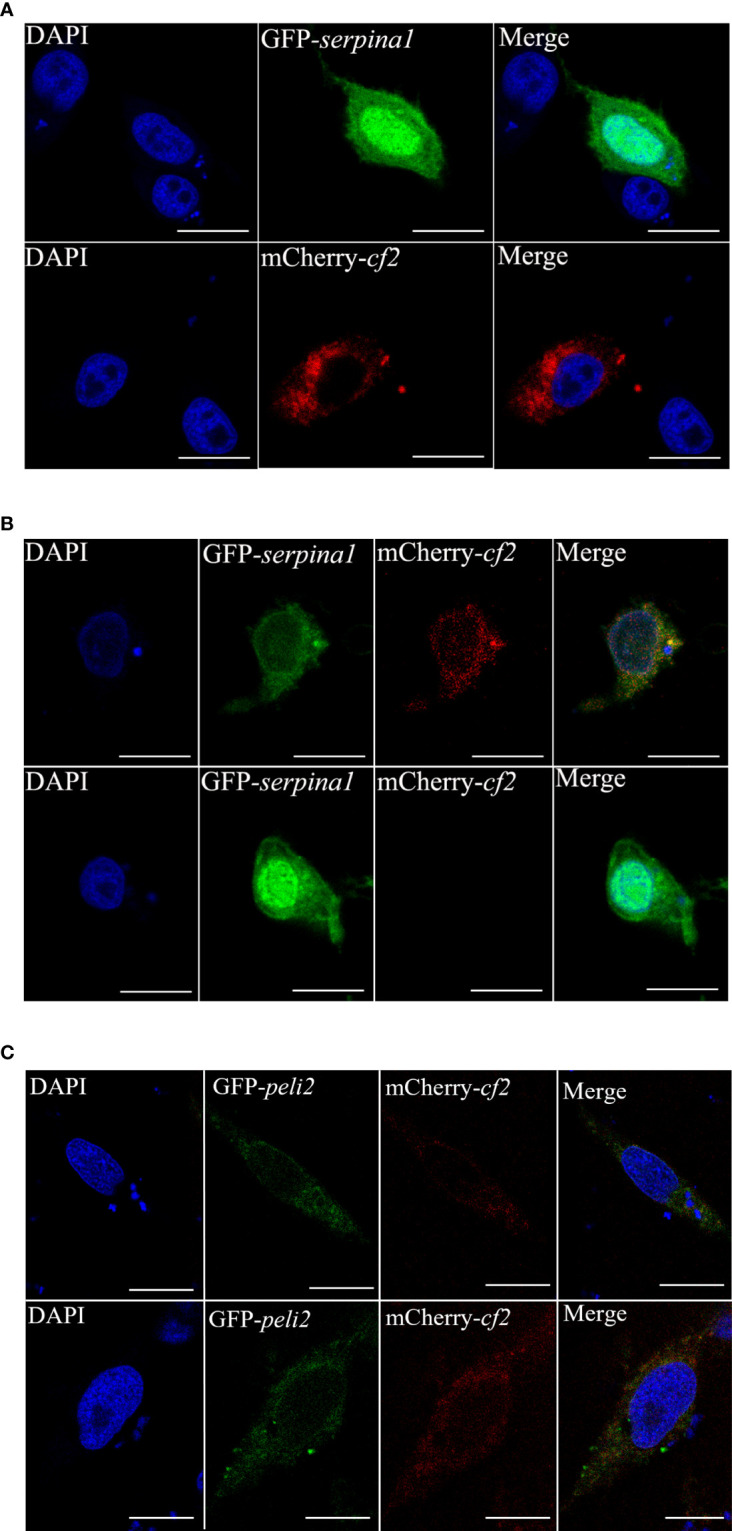
Subcellular co-localization of SERPINA1 and CF2 in CIK cells. **(A)** CIK cells expressing CF2 (top) or SERPINA1 (bottom) alone under confocal microscopy observation. The green fluorescent signal indicates SERPINA1 protein, the red fluorescent signal indicates CF2 protein, and the blue fluorescence indicates nucleus. **(B)** Fluorescence observation of CIK cells after co-expression of SERPINA1 and CF2 for 24 h (top) and 48 h (bottom). **(C)** Fluorescence observation of CIK cells after co-expression of PELI2 (GFP-tagged irrelevant protein) and CF2 for 24 h (top) and 48 h (bottom). The scale bar is 10 μm.

CIK cells were transfected with pEGFP-N3-*serpina1* and pCS2-mCherry-*cf2* alone, and cell fluorescence was observed using fluorescence confocal microscopy. When transfected with pEGFP-N3-*serpina1* alone, green fluorescence was observed throughout the cellular region, including the cytoplasm and nucleus. After transfection with pCS2-mCherry-*cf2* only, red fluorescence was observed in all cytoplasmic regions, except the nucleus (**Figure 4A**). pEGFP-N3-*serpina1* and pCS2-mCherry-*cf2* co-transfection showed colocalization of red and green fluorescence in the cytoplasmic region after 24 h. After 48 h, green fluorescence was observed in the entire cell area, including the cytoplasm and nucleus, and no red fluorescence was observed (**Figure 4B**). In the control group, the simultaneous presence of red fluorescence and green fluorescence during 24h – 48h could be observed without co-localization (**Figure 4C**). These results suggest that, when co-expressed, SERPINA1 and CF2 would first interact with each other, and then SERPINA1 would completely degrade CF2.

### SERPINA1 and CF2 overexpression affects the cellular inflammatory response and GCRV infection

**Figure 5:**
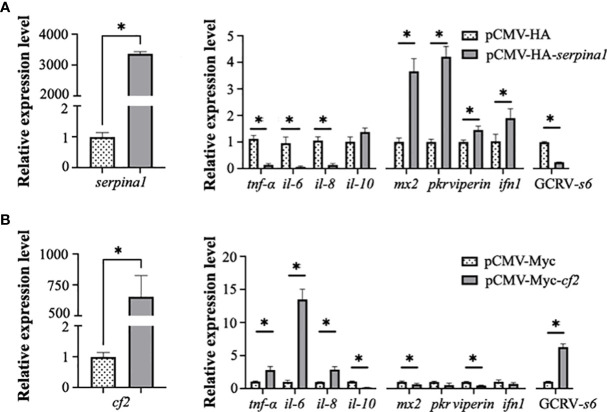
*Serpina1* and *cf2* overexpression alone affects the cellular inflammatory response and GCRV infection. **(A)** Changes in serpina1 expression level in cells were detected after *serpina1* overexpression (left). The relative expression levels of related cellular inflammatory factors, antiviral genes, and GCRV-*s6* were also detected (right). The horizontal coordinates are related genes and the vertical coordinates indicate the relative expression levels. **(B)** Changes in the expression of *cf2* in cells were detected after *cf2* overexpression. The relative expression levels of related cellular inflammatory factors, antiviral genes, and GCRV-*s6* were also detected. The horizontal coordinates are related genes and the vertical coordinates indicate the relative expression. The expression level of transfected null was set as “1” as control, and “*” indicates *p* < 0.05.

After overexpression of *serpina1* (3380-fold, *p* < 0.05) in transfected CIK cells, the expression of four inflammatory factors (*tnf-α*, *il-6*, and *il-8*) was significantly decreased compared with that in the control (0.12-fold; 0.07-fold; 0.12-fold, respectively; *p* < 0.05), and there was no significant change in expression of *il-10*. In addition, four antiviral protein-related genes (*mx2*, *pkr*, *viperin*, and *ifn1*) were examined, and their expression was found to be significantly elevated (3.63-fold, 4.20-fold, 1.45-fold, 1.85-fold, respectively; *p* < 0.05). Expression of the GCRV *s6* segment was significantly decreased (0.08-fold, *p* < 0.05) (**Figure 5A**). These results suggest that SERPINA1 increases the expression of antiviral genes and inhibits GCRV infection while suppressing the inflammatory response.

After overexpression of *cf2* (753.5-fold, *p* < 0.05) in transfected CIK cells, the expression of inflammatory factors *tnf-α*, *il-6*, and *il-8* was significantly higher (2.65-fold, 13.25-fold, 2.87-fold, respectively; *p* < 0.05), and the expression of *il-10* was significantly lower (0.16-fold, *p* < 0.05) than in the control cells. The expression of antiviral protein-related genes *mx2* and *viperin* was significantly decreased (0.62-fold and 0.45-fold, respectively; *p* < 0.05), whereas the expression of *pkr* and *ifn1* was not significantly changed. The expression of GCRV *s6* significantly increased (5.35-fold, *p* < 0.05) (**Figure 5B**). These results suggested that CF2 inhibits the expression of some antiviral genes and enhances GCRV infection in cells.

### *Serpina1* can affect virus proliferation in the presence of *cf2*

**Figure 6:**
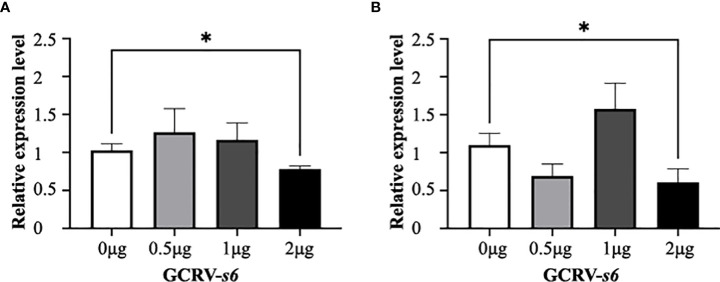
Expression changes of GCRV *s6* detected at different time points after co-expression of *serpina1* and *cf2*. **(A)** Relative expression levels of GCRV *s6* after 12 h of GCRV infection, when the transfection amount of *serpina1* gradually increased. **(B)** Relative expression levels of GCRV *s6* after 24 h of GCRV infection, when the transfection amount of *serpina1* gradually increased. The GCRV *s6* expression level, when transfected with 0 μg plasmid, was set as “1” as the control, and “*” indicates *p* < 0.05.

*Serpina1* and *cf2* were co-expressed in CIK cells. The transfection amount of pCMV-Myc-*cf2* plasmid was unchanged (2 μg), and the transfection amount of pCMV-HA-*serpina1* plasmid gradually increased in a gradient (0, 0.5, 1, and 2 μg). The results showed that the expression of GCRV *s6* did not change significantly in the three groups in which the transfection amount of pCMV-HA-*serpina1* plasmid increased from 0 μg to 1 μg after 12 h of GCRV infection, as compared with the control group; and decreased significantly in the 2 μg group (0.78-fold, *p* < 0.05) (**Figure 6A**). Similar results were observed for the samples after 24 h of GCRV infection, with no significant changes in GCRV *s6* expression in the three groups (0, 0.5, and 1 μg) and a significant decrease in GCRV *s6* expression in the 2 μg group (0.55-fold, *p* < 0.05) (**Figure 6B**). These results suggest that increasing SERPINA1 expression inhibits GCRV infection, regardless of the presence of CF2.

## Discussion

SERPINA1 is an acute response-phase protein synthesized mainly in the liver (31, 32). Grass carp *serpina1* is mainly expressed in hepatopancreas tissues, followed by immune tissues such as the head kidney and spleen. After GCRV infection, the response of grass carp *serpina1* was evident in immune tissues or virus contacting tissues, such as the head kidney, kidney, intestine, and gill, with peak expression on day 2 after infection, consistent with the characteristics of acute response phase proteins (33, 34). It has been hypothesized that SERPINA1 is involved in the early immune response during GCRV infection in grass carp.

This study found that grass carp SERPINA1 interacts with CF2 and can degrade it in a dose-dependent manner, and inhibit GCRV replication. The viral inhibitory function of SERPINA1 has been studied more frequently in the context of HIV (35–37). SERPINA1 directly interacts with the gp41 protein of HIV and thus inhibits its binding to the host CD4 co-receptor; thereby, inhibiting viral infection (38). It also inhibits gp120, thus preventing the maturation of the key HIV morphological viral proteins (36). There is no direct evidence of protein-interacting effects of SERPINA1 on prothrombin (13, 39). There are no reports on the involvement of SERPINA1 in antiviral immune processes in fish. In this study, we found that overexpression of grass carp *serpina1* significantly increased the expression levels of antiviral-related genes *mx2*, *pkr*, *viperin*, and *ifn1* in CIK cells. This study suggests that fish serine protease inhibitors can also inhibit viral replication because fish SERPINA1 can degrade prothrombin. Whether the interaction between SERPINA1 and CF2 is direct or indirect requires further experimental verification.

When infected with GCRV, *cf2* overexpression in CIK cells was found to facilitate viral replication. Activation of thrombin has been found to enhance viral infection in several studies (40), and type I and II herpes simplex viruses use protease-activated receptor 1 (PAR1) to stimulate thrombin production and infect cells (41). During the infection of A549 cells with the respiratory syncytial virus, thrombin enhances virus-cell fusion and facilitates virus entry through the syncytial pathway (42). In addition, thrombin in host cells cleaves the protein encoded by the *pORF1* gene of the hepatitis E virus, which is necessary for the replication of the virus (43). During viral infections that cause bleeding symptoms, viruses can activate thrombin followed by platelets, causing intravascular coagulation in the organism (44, 45). Most grass carp infected with GCRV showed fatal hemorrhagic symptoms; however, the host coagulation or anticoagulation mechanism caused by GCRV infection is unclear. Combined with the *cf2* overexpression results of this study, we hypothesized that the GCRV may induce excessive production of coagulation factors in the body of fish to facilitate its replication, leading to thrombus formation and disruption of blood homeostasis, thus producing hemorrhagic symptoms.

This study also found that overexpression of *serpina1* in CIK cells effectively inhibited the expression of the inflammatory cytokines *tnf-α*, *il-6*, and *il-8*, whereas the overexpression of *cf2* had almost the exact opposite effect. The acute-phase response to viral infection usually manifests as pro-inflammatory changes and is associated with viral clearance (33, 46). As the infection progresses, the virus gradually compromises the circulatory system, triggering symptoms of dysfunction and inflammatory lesions in various organs. Previous studies have shown that during an inflammatory response, SERPINA1 reduces the expression of pro-inflammatory cytokines and chemokines induced by neutrophils and inhibits the inflammatory response of macrophages (47, 48). Furthermore, there is an extensive link between inflammation and coagulation, with organismal inflammation-activating thrombin, which in turn affects inflammatory activity (49–51). Combined with the results of CF2 degradation by SERPINA1 in this study, it is hypothesized that SERPINA1 may inhibit the inflammatory response induced by CF2 after GCRV infection of grass carp on the one hand, and attenuate the effect of GCRV use of CF2 to enhance infection on the other. A balanced relationship needs to be maintained between coagulation reaction and antiviral reaction. Once the balance is broken, it will endanger the life of grass carp. It has been shown that fish can influence viral infection by regulating apoptosis or the interferon pathway (52, 53). This study is the first to report SERPINA1 inhibition of GCRV infection through prothrombin, which provides new evidence for further study of the antiviral function of fish SERPINs.

## Data availability statement

The original contributions presented in the study are publicly available. The mass spectrometry proteomics data have been deposited to the ProteomeXchange Consortium via the PRIDE (54) partner repository with the dataset identifier PXD036356.

## Ethics statement

The animal study was reviewed and approved by Academic Committee of the Institute of Hydrobiology, Chinese Academy of Sciences.

## Author contributions

RH, LC, and YYaL supervised the overall project and designed the experiments. YYaL and LC performed the experiments, analyzed the data, and wrote the manuscript. YYuL, CY, and BG helped to perform experiments and analyzed the data. YML and LL were responsible for providing experimental fish and sampling. YW and ZZ provided advice on manuscript writing. All authors reviewed the manuscript. All authors contributed to the article and approved the submitted version.

## Funding

This work was supported by the National Natural Science Foundation of China (31972788 and 31721005) and the State Key Laboratory of Freshwater Ecology and Biotechnology (2019FBZ05, 2021FB11).

## Acknowledgments

We thank Dr. Fang Zhou (Institute of Hydrobiology, Chinese Academy of Sciences) for assistance with confocal microscopy analysis.

## Conflict of interest

The authors declare that the research was conducted in the absence of any commercial or financial relationships that could be construed as a potential conflict of interest.

## Publisher’s note

All claims expressed in this article are solely those of the authors and do not necessarily represent those of their affiliated organizations, or those of the publisher, the editors and the reviewers. Any product that may be evaluated in this article, or claim that may be made by its manufacturer, is not guaranteed or endorsed by the publisher.

## Supplementary material

Supplementary Table 1 Primers for qPCR and plasmid constructions.

Supplementary Table 2 Mass spectrometry analysis data of screening SERPINA1 interacting proteins.

Supplementary Figure 1 Validation of SERPINA1 antibody.

## References

[1] FAO. The State of World Fisheries and Aquaculture 2020. Sustainability in action. (Rome FAO) (2022). doi: 10.4060/ca9229en

[2] Rangel AAC Rockemann DD Hetrick FM Samal SK. Identification of grass carp haemorrhage virus as a new genogroup of aquareovirus. J Gen Virol (1999) 80(9):2399–402. doi: 10.1099/0022-1317-80-9-2399 10501493

[3] Jiang Y. Hemorrhagic disease of grass carp: Status of outbreaks, diagnosis, surveillance, and research. Israeli J Aquacult - Bamidgeh (2009) 61:188–97. doi: 10.46989/001c.20560

[4] Qiu D-K Zhao Z Ma R Guo Z-R Jia Y-J Zhang C. Antigen epitope screening of grass carp reovirus and its protectively immunity assessment for grass carp. Aquaculture (2020) 515:734550. doi: 10.1016/j.aquaculture.2019.734550

[5] Heit C Jackson BC McAndrews M Wright MW Thompson DC Silverman GA. Update of the human and mouse SERPIN gene superfamily. Hum Genomics (2013) 7(1):14. doi: 10.1186/1479-7364-7-22 24172014 PMC3880077

[6] Huntington JA Read RJ Carrell RW. Structure of a serpin-protease complex shows inhibition by deformation. Nature (2000) 407(6806):923–6. doi: 10.1038/35038119 11057674

[7] Khan MS Singh P Azhar A Naseem A Rashid Q Kabir MA. Serpin inhibition mechanism: A delicate balance between native metastable state and polymerization. J Amino Acids (2011) 2011:606797. doi: 10.4061/2011/606797 22312466 PMC3268027

[8] Law RH Zhang Q McGowan S Buckle AM Silverman GA Wong W. An overview of the serpin superfamily. Genome Biol (2006) 7(5):216. doi: 10.1186/gb-2006-7-5-216 16737556 PMC1779521

[9] Silverman GA Bird PI Carrell RW Church FC Coughlin PB Gettins PG. The serpins are an expanding superfamily of structurally similar but functionally diverse proteins. Evolution, mechanism of inhibition, novel functions, and a revised nomenclature. J Biol Chem (2001) 276(36):33293–6. doi: 10.1074/jbc.R100016200 11435447

[10] Yamaya M Shimotai Y Hatachi Y Lusamba Kalonji N Tando Y Kitajima Y. The serine protease inhibitor camostat inhibits influenza virus replication and cytokine production in primary cultures of human tracheal epithelial cells. Pulm Pharmacol Ther (2015) 33:66–74. doi: 10.1016/j.pupt.2015.07.001 26166259 PMC7110702

[11] Gong D Farley K White M Hartshorn KL Benarafa C Remold-O'Donnell E. Critical role of serpinB1 in regulating inflammatory responses in pulmonary influenza infection. J Infect Dis (2011) 204(4):592–600. doi: 10.1093/infdis/jir352 21791661 PMC3144176

[12] Burgener SS Leborgne NGF Snipas SJ Salvesen GS Bird PI Benarafa C. Cathepsin G inhibition by Serpinb1 and Serpinb6 prevents programmed necrosis in neutrophils and monocytes and reduces GSDMD-driven inflammation. Cell Rep (2019) 27(12):3646–56.e5. doi: 10.1016/j.celrep.2019.05.065 31216481 PMC7350907

[13] Whitney JB Asmal M Geiben-Lynn R. Serpin induced antiviral activity of prostaglandin synthetase-2 against HIV-1 replication. PLoS One (2011) 6(4):e18589. doi: 10.1371/journal.pone.0018589 21533265 PMC3075258

[14] Mangan MS Kaiserman D Bird PI. The role of serpins in vertebrate immunity. Tissue Antigens (2008) 72(1):1–10. doi: 10.1111/j.1399-0039.2008.01059.x 18498291

[15] Hurley K Lacey N O'Dwyer CA Bergin DA McElvaney OJ O'Brien ME. Alpha-1 antitrypsin augmentation therapy corrects accelerated neutrophil apoptosis in deficient individuals. J Immunol 193(8):3978–9. doi: 10.4049/jimmunol.1400132 25217166

[16] Hurley K Lacey N O'Dwyer CA Bergin DA McElvaney OJ O'Brien ME. Alpha-1 antitrypsin augmentation therapy corrects accelerated neutrophil apoptosis in deficient individuals. J Immunol (2014) 193(8):3978–91. doi: 10.4049/jimmunol.1400132 25217166

[17] Song S. Alpha-1 antitrypsin therapy for autoimmune disorders. Chronic Obstr Pulm Dis (2018) 5(4):289–301. doi: 10.15326/jcopdf.5.4.2018.0131 30723786 PMC6361478

[18] Kaneva MK Muley MM Krustev E Reid AR Souza PR Dell'Accio F. Alpha-1-antitrypsin reduces inflammation and exerts chondroprotection in arthritis. FASEB J (2021) 35(5):e21472. doi: 10.1096/fj.202001801R 33788977

[19] Janciauskiene S Welte T. Well-known and less well-known functions of alpha-1 antitrypsin. its role in chronic obstructive pulmonary disease and other disease developments. Ann Am Thorac Soc (2016) 13 Suppl 4:S280–8. doi: 10.1513/AnnalsATS.201507-468KV 27564662

[20] Nita IM Serapinas D Janciauskiene SM. alpha1-antitrypsin regulates CD14 expression and soluble CD14 levels in human monocytes in vitro. Int J Biochem Cell Biol (2007) 39(6):1165–76. doi: 10.1016/j.biocel.2007.02.017 17448722

[21] Janciauskiene S Larsson S Larsson P Virtala R Jansson L Stevens T. Inhibition of lipopolysaccharide-mediated human monocyte activation, *in vitro*, by alpha1-antitrypsin. Biochem Biophys Res Commun (2004) 321(3):592–600. doi: 10.1016/j.bbrc.2004.06.123 15358147

[22] Shapiro L Pott B Ralston H. Alpha-1-antitrypsin inhibits human immunodeficiency virus type 1. FASEB J (2001) 15(1):115–22. doi: 10.1096/fj.00-0311com 11149899

[23] Wanner A Arce AD Pardee E. Novel therapeutic uses of alpha-1 antitrypsin: a window to the future. COPD (2012) 9(6):583–8. doi: 10.3109/15412555.2012.717125 23205701

[24] Shi M Huang R Du F Pei Y Liao L Zhu Z. RNA-Seq profiles from grass carp tissues after reovirus (GCRV) infection based on singular and modular enrichment analyses. Mol Immunol (2014) 61(1):44–53. doi: 10.1016/j.molimm.2014.05.004 24865419

[25] Chu P He L Xiong L Luo L Huang R Liao L. Molecular cloning, expression analysis and localization pattern of the MST family in grass carp (Ctenopharyngodon idella). Fish Shellfish Immunol (2018) 76:316–23. doi: 10.1016/j.fsi.2018.03.021 29550601

[26] Wang Y Lu Y Zhang Y Ning Z Li Y Zhao Q. The draft genome of the grass carp (Ctenopharyngodon idellus) provides insights into its evolution and vegetarian adaptation. Nat Genet (2015) 47(6):625–31. doi: 10.1038/ng.3280 25938946

[27] Livak KJ Schmittgen TD. Analysis of relative gene expression data using real-time quantitative PCR and the 2(-delta delta C(T)) method. Methods (2001) 25(4):402–8. doi: 10.1006/meth.2001.1262 11846609

[28] Andrews GL Simons BL Young JB Hawkridge AM Muddiman DC. Performance characteristics of a new hybrid quadrupole time-of-flight tandem mass spectrometer (TripleTOF 5600). Anal Chem (2011) 83(13):5442–6. doi: 10.1021/ac200812d PMC3138073 21619048

[29] Shilov IV Seymour SL Patel AA Loboda A Tang WH Keating SP. The paragon algorithm, a next generation search engine that uses sequence temperature values and feature probabilities to identify peptides from tandem mass spectra*. Mol Cell Proteomics (2007) 6(9):1638–55. doi: 10.1074/mcp.T600050-MCP200 17533153

[30] Kalz J ten Cate H Spronk HM. Thrombin generation and atherosclerosis. J Thromb Thrombolysis (2014) 37(1):45–55. doi: 10.1007/s11239-013-1026-5 24241912

[31] Ehlers MR. Immune-modulating effects of alpha-1 antitrypsin. Biol Chem (2014) 395(10):1187–93. doi: 10.1515/hsz-2014-0161 PMC4237306 24854541

[32] Nallagangula KS Shashidhar KN Lakshmaiah V Muninarayana C. Cirrhosis of liver: Interference of serpins in quantification of SERPINA4 - a preliminary study. Pract Lab Med (2017) 9:53–7. doi: 10.1016/j.plabm.2017.10.002 PMC5683666 29159256

[33] Cem G Kushner I. Acute-phase proteins and other systemic responses to inflammation. New Engl J Med (1999) 340:6. doi: 10.1056/NEJM199902113400607 9971870

[34] Kaner Z Ochayon DE Shahaf G Baranovski BM Bahar N Mizrahi M. Acute phase protein alpha1-antitrypsin reduces the bacterial burden in mice by selective modulation of innate cell responses. J Infect Dis (2015) 211(9):1489–98. doi: 10.1093/infdis/jiu620 25389308

[35] Congote LF. The c-terminal 26-residue peptide of serpin A1 is an inhibitor of HIV-1. Biochem Biophys Res Commun (2006) 343(2):617–22. doi: 10.1016/j.bbrc.2006.02.190 16554023

[36] Cordelier P Strayer DS. Mechanisms of α1-antitrypsin inhibition of cellular serine proteases and HIV-1 protease that are essential for HIV-1 morphogenesis. Biochim Biophys Acta (BBA) - Mol Basis Disease (2003) 1638(3):197–207. doi: 10.1016/S0925-4439(03)00084-X 12878320

[37] Cordelier P Strayer DS. Conditional expression of alpha1-antitrypsin delivered by recombinant SV40 vectors protects lymphocytes against HIV. Gene Ther (2003) 10(26):2153–6. doi: 10.1038/sj.gt.3302113 14625571

[38] Zhou X Liu Z Zhang J Adelsberger JW Yang J Burton GF. Alpha-1-antitrypsin interacts with gp41 to block HIV-1 entry into CD4+ T lymphocytes. BMC Microbiol (2016) 16(1):172. doi: 10.1186/s12866-016-0751-2 27473095 PMC4966588

[39] Rau JC Beaulieu LM Huntington JA Church FC. Serpins in thrombosis, hemostasis and fibrinolysis. J Thromb Haemost (2007) 5 Suppl 1:102–15. doi: 10.1111/j.1538-7836.2007.02516.x PMC2670448 17635716

[40] Laine O Mäkelä S Mustonen J Huhtala H Szanto T Vaheri A. Enhanced thrombin formation and fibrinolysis during acute puumala hantavirus infection. Thromb Res (2010) 126(2):154–8. doi: 10.1016/j.thromres.2010.05.025 20579693

[41] Sutherland MR Friedman HM Pryzdial EL. Thrombin enhances herpes simplex virus infection of cells involving protease-activated receptor 1. J Thromb Haemost (2007) 5(5):1055–61. doi: 10.1111/j.1538-7836.2007.02441.x 17461934

[42] Dubovi EJ Geratz JD Tidwell RR. Enhancement of respiratory syncytial virus-induced cytopathology by trypsin, thrombin, and plasmin. Infect Immun (1983) 40(1):351–8. doi: 10.1128/iai.40.1.351-358.1983 PMC264855 6219957

[43] Kanade GD Pingale KD Karpe YA. Activities of thrombin and factor xa are essential for replication of hepatitis e virus and are possibly implicated in ORF1 polyprotein processing. J Virol (2018) 92(6):2854–63. doi: 10.1128/JVI.01853-17 PMC5827406 29321328

[44] Boilard E Pare G Rousseau M Cloutier N Dubuc I Levesque T. Influenza virus H1N1 activates platelets through FcgammaRIIA signaling and thrombin generation. Blood (2014) 123(18):2854–63. doi: 10.1182/blood-2013-07-515536 24665136

[45] Visser MR Tracy PB Vercellotti GM Goodman JL White JG Jacob HS. Enhanced thrombin generation and platelet binding on herpes simplex virus-infected endothelium. Proc Natl Acad Sci U S A (1988) 85(21):8227–30. doi: 10.1073/pnas.85.21.8227 PMC282402 2847155

[46] Tisoncik JR Korth MJ Simmons CP Farrar J Martin TR Katze MG. Into the eye of the cytokine storm. Microbiol Mol Biol Rev (2012) 76(1):16–32. doi: 10.1128/MMBR.05015-11 22390970 PMC3294426

[47] Shahaf G Moser H Ozeri E Mizrahi M Abecassis A Lewis EC. Alpha-1-antitrypsin gene delivery reduces inflammation, increases T-regulatory cell population size and prevents islet allograft rejection. Mol Med (2011) 17(9-10):1000–11. doi: 10.2119/molmed.2011.00145 PMC3188864 21670848

[48] Jonigk D Al-Omari M Maegel L Muller M Izykowski N Hong J. Anti-inflammatory and immunomodulatory properties of alpha1-antitrypsin without inhibition of elastase. Proc Natl Acad Sci U S A (2013) 110(37):15007–12. doi: 10.1073/pnas.1309648110 PMC3773761 23975926

[49] Levi M van der Poll T Buller HR. Bidirectional relation between inflammation and coagulation. Circulation (2004) 109(22):2698–704. doi: 10.1161/01.CIR.0000131660.51520.9A 15184294

[50] Chen D Dorling A. Critical roles for thrombin in acute and chronic inflammation. J Thromb Haemost (2009) 7 Suppl 1:122–6. doi: 10.1111/j.1538-7836.2009.03413.x 19630783

[51] Huerta-Zepeda A Cabello-Gutierrez C Cime-Castillo J Monroy-Martinez V Manjarrez-Zavala ME Gutierrez-Rodriguez M. Crosstalk between coagulation and inflammation during dengue virus infection. Thromb Haemost (2008) 99(5):936–43. doi: 10.1160/TH07-08-0483 18449425

[52] Xu X Li M Deng Z Hu J Jiang Z Liu Y. Grass carp (Ctenopharyngodon idellus) NIMA-related kinase 6 blocks dsRNA-induced IFN I response by targeting IRF3. Front Immunol (2020) 11:597775. doi: 10.3389/fimmu.2020.597775 33488591 PMC7820699

[53] Xu X Li M Li D Jiang Z Liu C Shi X. Identification of the SAMHD1 gene in grass carp and its roles in inducing apoptosis and inhibiting GCRV proliferation. Fish Shellfish Immunol (2019) 88:606–18. doi: 10.1016/j.fsi.2019.03.028 30885743

[54] Perez-Riverol Y bai J Bandla C Garcia-Seisdedos D Hewapathirana S Kamatchinathan S. The PRIDE database resources in 2022: A hub for mass spectrometry-based proteomics evidences. Nucleic Acids Res (2022) 50(D1):D543–52. doi: 10.1093/nar/gkab1038 PMC8728295 34723319

