# Supplementary figures and images for: Grass carp SERPINA1 inhibits GCRV infection through degrading CF2
Source: Front Immunol. 2022 Sep 8;13:969517. doi: 10.3389/fimmu.2022.969517 (PMC9494734; doi:10.3389/fimmu.2022.969517)

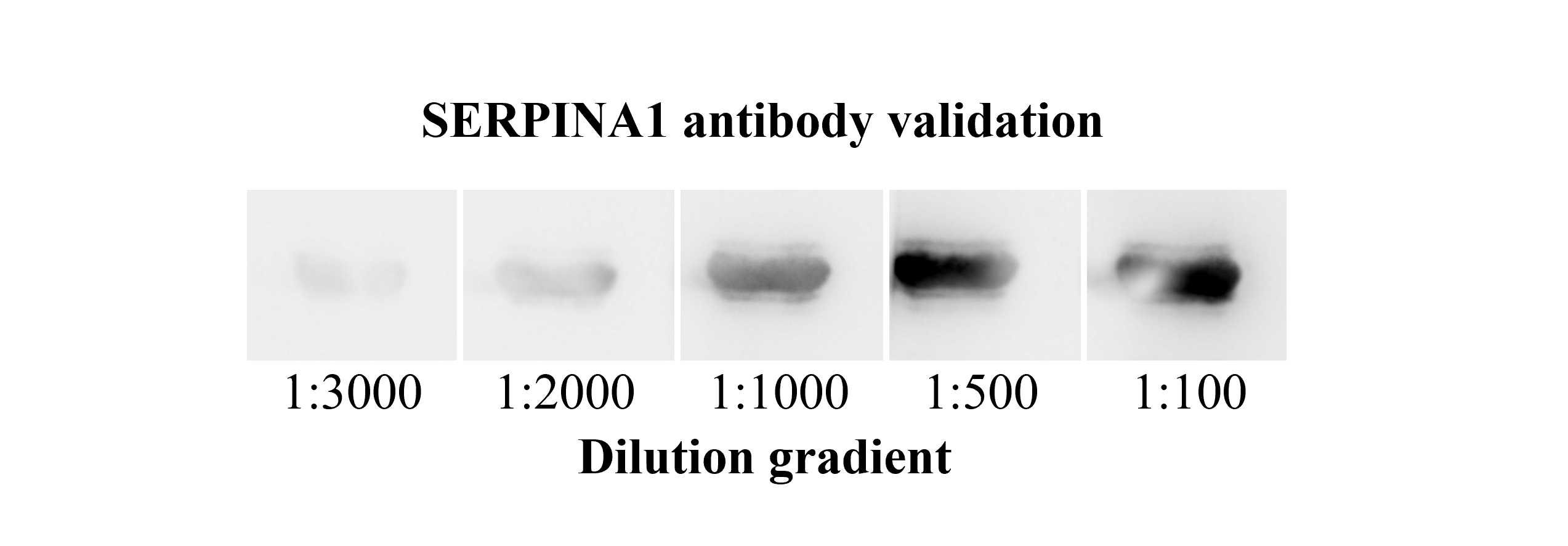

Supplement: Supplementary Figure 1 — Validation of SERPINA1 antibody. [file Image_1.tif]
